# The Infuence of Salicin on Rheological and Film-Forming Properties of Collagen

**DOI:** 10.3390/molecules26061661

**Published:** 2021-03-16

**Authors:** Katarzyna Adamiak, Katarzyna Lewandowska, Alina Sionkowska

**Affiliations:** 1 Department of Biomaterials and Cosmetics Chemistry, Faculty of Chemistry, Nicolaus Copernicus University in Torun, Gagarin 7 Street, 87-100 Torun, Poland; kadamiak@wellu.eu (K.A.); reol@umk.pl (K.L.); 2 WellU sp.z.o.o, Wielkopolska 280, 81-531 Gdynia, Poland

**Keywords:** collagen, salicin, cross-linking, biomaterials, incorporation, collagen films

## Abstract

Collagen films are widely used as adhesives in medicine and cosmetology. However, its properties require modification. In this work, the influence of salicin on the properties of collagen solution and films was studied. Collagen was extracted from silver carp skin. The rheological properties of collagen solutions with and without salicin were characterized by steady shear tests. Thin collagen films were prepared by solvent evaporation. The structure of films was researched using infrared spectroscopy. The surface properties of films were investigated using Atomic Force Microscopy (AFM). Mechanical properties were measured as well. It was found that the addition of salicin modified the roughness of collagen films and their mechanical and rheological properties. The above-mentioned parameters are very important in potential applications of collagen films containing salicin.

## 1. Introduction

Collagen-based biomaterials are widely used in the pharmacological, medical, and cosmetological fields due to their biocompatibility, non-toxicity, and natural origin [1,2,3]. Collagen type I which is widely applied in the above-mentioned fields is composed of three polypeptide chains coiled around each other. A particular feature of collagen is the presence of specific amino acids: glycine, proline, and hydroxyproline. The molecule is held together mainly by hydrogen bindings among groups such as CO and NH. The maintenance of this protein structure in its mature form is ensured also by covalent bonds [4,5]. Triple helices are being arranged to flexible fibrils that can be further cross-linked to improve the mechanical resistance of the collagen material [6,7]. Individual triple-helical domain pertains further than 95% of the molecule in the classic fibril-forming collagens. In some of the collagens, it occurs that the triple-helical domain constitutes only a fraction of the molecule’s mass, but the triple-helices are multiple [8,9,10,11,12].

Collagen is considered as a proper biomaterial for tissue engineering, wound healing, drug delivery, and cosmetics, mainly because the green alternative industry seeks non-toxic and biodegradable polymers [13]. Collagen is a natural polymer and may promote the migration of cells forming an extracellular matrix (ECM) accelerating the tissue regenerative process [14]. There are 29 types of collagen that have been characterized so far. Type I represents the most ordered structure, which provides high tensile strength [15,16,17]. It is also the most common type of protein in mammals. It builds ligaments, tendons, skin, and bones. Every tissue has distinctive strength and elasticity requirements which shows a diversity of collagen as a matrix [18]. Collagen constitutes a natural binding matrix in the tissue regeneration and reconstruction process [19]. Incorporating the proper substances into the collagen matrix can lead to the enhancement of material properties. However, the origin of collagen is very important for its future uses. The utilization of marine-based collagen is growing fast due to its unique properties in comparison with mammalian-based collagen such as no risk of transmitting diseases and a lack of religious constraints. The extraction of collagen from marine sources can be a cost-effective process and may lead to low molecular weight collagen with good biocompatibility and absorbability by the human body [20,21]. In particular, the utilization of silver carp collagen is growing fast due to the fact of its relatively high denaturation temperature in comparison to other kinds of fish collagens [21].

The proper substances for incorporation into collagen can be several plant extracts, including willow bark extract which contains salicin. Willow bark extract is eminently efficient in the treatment of diseases like rheumatoid arthritis [22,23], painful mobility disorders [24], osteoarthritis [25,26,27], and other inflammatory afflictions. Anti-inflammatory properties of salicin and its derivatives in willow bark extract include natural pro-drugs of salicylates [24]. Salicin is the Latin word for willow, salix. The willow is a part of the *Salicaceae* family that is represented by species: *Salix alba* L., *Salix purpurea* L., *Salix daphnoides Vill*., *Salix fragilis* L., *Salix nigra Marshall*. These species have the highest quantity of salicylate precursors [28]. The source of salicin constitutes willow bark and leaves. Salicin is a white substance with a bitter taste in the powder form, which can also be named as 2-(hydroxymethyl)phenyl; β-d-glucopyranoside; 2-(hydroxymethyl)phenyl; salicine; 2-(hydroxymethyl)phenyl-β-d-glucopyranoside; salicoside; saligenin-β-d-glucopyranoside and salicyl alcohol glucoside [29]. Salicin is often used as a biological activity marker of willow bark [30]. The raw material is also rich in polyphenols, proanthocyanidins, tanins, and flavonoids (for example eriodictyol, isosalipurpuroside, naringenin) [24,31,32]. The willow bark extract has anti-inflammatory, antipyretic, antirheumatic, antigesic, and antiseptic properties [33]. The latest research has shown that salicin from willow bark extract prevents collagen type II from degradation by matrix metalloproteinases MMP-1/3/13, such as MMP-1, MMP-3, and MMP-13, which can be an important factor in collagen matrix amelioration as a biomaterial [25]. In cosmetic applications, salicin exhibits anti-irritative properties in topical dermatological applications [34]. Salicin shows also an anti-aging effect on the skin [35].

The aim of this work was the preparation of silver carp collagen films incorporated with salicin. The surface and mechanical properties of the new materials were studied. The above-mentioned properties are very important for the application of collagen films containing salicin as adhesives.

## 2. Materials and Methods

### 2.1. Materials

Fish collagen was supplied by WellU sp.z.o.o. (Gdynia, Poland) and willow bark dry extract containing 5% of salicin by Greenvit (Zambrow, Poland). For collagen extraction, the skin of silver carp was used because of its relatively high denaturation temperature [36,37].

### 2.2. Mixture Preparation

#### 2.2.1. Collagen Solution Preparation

Collagen was isolated from silver carp skin. The skin scales and other elements were removed manually and cleaned with chilled tap water to get rid of the adhering tissues. Then, the material was sanitized with a 3% hydrogen peroxide water solution. The residues were rinsed off. The purified skin was placed in 0.1 M acetic acid solution and left for 3 days to extract the collagenous proteins. The obtained solution was pressed through the properly chosen material, which allowed for collagen separation [37]. Then the samples were lyophilized (ALPHA 1-2 LDplus, CHRIST, Osterode, Germany; −20 °C, 100 Pa, 48 h). After lyophilization, collagen solution was prepared by diluting lyophilizate in 0.1 M acetic acid at the 5 mg/mL concentration.

#### 2.2.2. Willow Bark Solution Preparation

At the first stage, the dry willow bark extract containing 5% of salicin was weight out in 0.4974 g, then it was moved quantitatively into a 10 mL volumetric flask, then refilled to 10 mL with water and mixed to absolute dissolution. The next stage was transferring the collagen solution to a 25 mL volumetric flask and the addition of 1 mL of willow bark water solution prepared in the previous stage. The amount of salicin in collagen was 1.98%.

### 2.3. Film-Forming Process

Collagen solution and mixed collagen-salicin solution were put into the proper plastic plates after checking the proper surface level. The plastic plates were covered proportionally with the solutions. Samples were left to dry for 6 days. Then collagen films were cautiously peeled off and examined.

### 2.4. Infrared Spectroscopy (IR)

IR spectra were registered using Nicolet iS10 spectrophotometer equipped with an ATR device with a germanium crystal (Thermo Fisher Scientific, Waltham, MA, USA). All the spectra were recorded with a resolution of 4 cm^−1^ with 64 scans. The spectra were evaluated in the range of 400–4000 cm^−1^. The data were collected and plotted using the Omnic Spectra 2009 program supplied by the manufacturer. 

### 2.5. Scanning Electron Microscopy (SEM)

Samples were imaged by using Scanning Electron Microscope (SEM), (LEO Electron Microscopy Ltd., Cambridge, England, UK). Samples were covered by gold to form the conductive surface for the electron beam interaction. Micrographs of all samples were taken at 300× magnification.

### 2.6. Energy-Dispersive X-ray Spectroscopy (EDX)

Energy-dispersive X-ray spectroscopy (EDX) was used to assess the elemental composition of a material (Energy Dispersive X-ray Spectrometer EDX Quantax 200 with detector XFlask 4010, Bruker, AXC, Berlin, Germany).

### 2.7. Atomic Force Microscopy (AFM)

The surface structure of collagen/salicin materials was analyzed based on Atomic Force Microscope pictures obtained by MultiMode Scanning Probe Microscope Nanoscope IIIa (Digital Instruments Veeco Metrology Group, Santa Barbara, CA, USA) operating in the tapping mode, in air, at room temperature. Surface images were acquired at fixed resolution (512 × 512 data points) with a scan rate of 1.97 Hz. Silicon tips with a spring constant of 2–10 N/m were used. Roughness parameters were calculated from 10 μm × 10 μm scanned area using Nanoscope software (Bruker Optoc GmbH, Ettlingen, Germany).

### 2.8. Contact Angle Measurements

The contact angles of two liquids: glycerol (G) and diiodomethane (D) on the surface of collagen films were measured at constant temperature (22 °C) using a goniometer equipped with the system of drop-shape analysis (DSA 10 Control Unit, Krüss, Hamburg, Germany). The embedded drop procedure was used as follows: a drop of glycerine or diiodomethane was placed onto a collagen film surface with a microsyringe and the value of contact angle was determined based on the obtained image of the liquid drop. The results, which were an average of 6 measurements (the deviation from the average was within ±2°), allowed to calculate the surface free energy.

The surface free energy was calculated using the Owens–Wendt method, as this method is commonly used for the calculation of polar and dispersive components of surface free energy for polymers [38].

### 2.9. Mechanical Properties

The shaped pieces cut from collagen and collagen-salicin films were prepared using manual press Optimum DDP10 (Optimum Maschinen Germany GmbH, Hallstadt, Germany).

Mechanical properties of collagen and collagen/salicin films such as Young Modulus and tensile strength were tested using Zwick & Roell Z.0.5 (Zwick&Roell Group, Ulm, Germany) testing machine in a dry condition at room temperature. Parameters of the testing program: 200 mm/min speed starting position, 0.1 N initial force, 5 mm/min the speed of the initial force. 10 samples of each kind of film were measured.

### 2.10. Steady Shear Flow Properties

Collagen and collagen/salicin solutions were prepared in the same way as in the Section 2.2. Flow measurements were conducted using a Bohlin Visco 88 rotary viscometer (Malvern Panalytical, Malvern, UK) equipped with concentric cylinders (22 °C and 26 °C) adequately for collagen and collagen/salicin mixture. The Ostwald de Waele model and Cross model were applied to describe the steady shear flow properties of collagen solutions [37].

## 3. Results and Discussion

### 3.1. Physicochemical Properties

**Figure 1:**
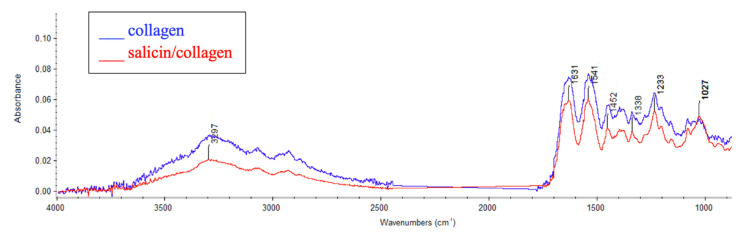
Infrared (IR) spectra of salicin/collagen film and collagen film (control) from 4000 to 500 cm^−1^.

To assess the interaction between salicin and collagen and to confirm the presence of salicin in collagen films the IR spectra were registered. The positions of IR bands have been discussed (Table 1). The IR spectra have been shown in Figure 1.

The broad band at around 3500–2500 cm^−1^ can be assigned to amide A (N-H stretching) and OH in collagen [38,39]. Distinctive bands for collagen are also visible at 1631 cm^−1^ for amide I, which indicates C=O stretching, at 1541 cm^−1^ for amide II (N-H binding), and 1233 cm^−1^ for amide III (C-N bending).

Salicin is an aryl β-d-glucoside and constitutes an aromatic primary alcohol-salicyl alcohol that has the phenolic hydrogen replaced by a β-d-glucosyl residue. It also belongs to benzyl alcohols [40].

As we can see in Figure 1 for the collagen/salicin sample the following bands can be observed: O–H groups at around 3200–2600 cm^−1^ and strong C–O stretching at 1027 cm^−1^, because salicin as a saligenin glucoside contains glucoside bonds. For collagen with the addition of salicin the shift of amide A band was observed (from 3291 cm^−1^ to 3297 cm^−1^). It may suggest the interaction between collagen and salicin via hydrogen bonds.

### 3.2. Morphological Properties

**Figure 2:**
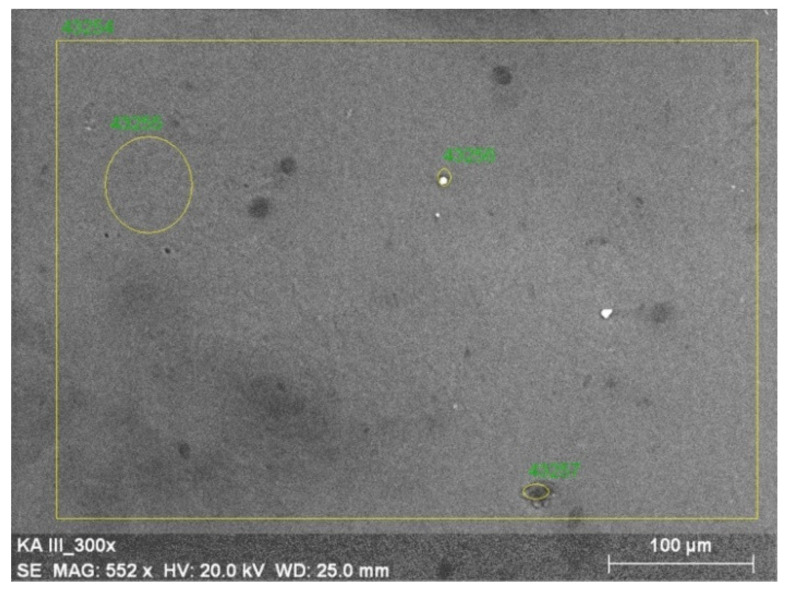
SEM image of salicin/collagen film.

To investigate the surface structure of the film SEM imaging and EDX analysis were performed. In Figure 2 the SEM image has been shown. As one can see the membrane exhibits a smooth, compact, heteroclite, and flattened surface. The packaging of collagen fibrils is loose.

**Figure 3:**
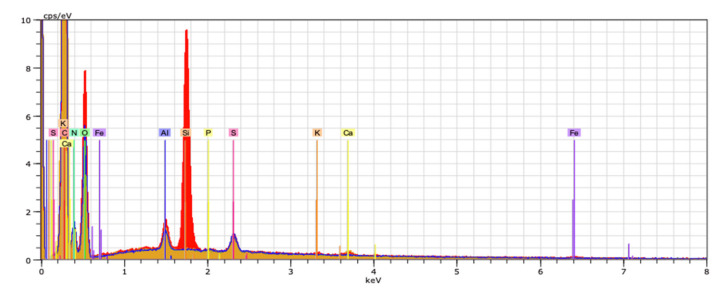
The percentage element range in sample collagen/salicin.

EDX spectroscopy was performed to assess the elemental composition and topography of the material. The percentage elements range in collagen/salicin sample is visualized in Figure 3 in the adequate voltage attribution counted in seconds per electron-volt at accelerating voltage range (keV) for EDX analysis.

The mean value of the mass percentage of the C element in the sample was 41.09%, whereas for N it was 18.07%, for O it was 38.44%, which indicates the presence of collagen. Similar results have been obtained for collagen films, however, the presence of salicin in collagen has been proved by IR spectroscopy (C-O stretching at 1027 cm^−1^ of glucoside bonds in salicin).

### 3.3. Atomic Force Microscopy (AFM)

**Figure 4:**
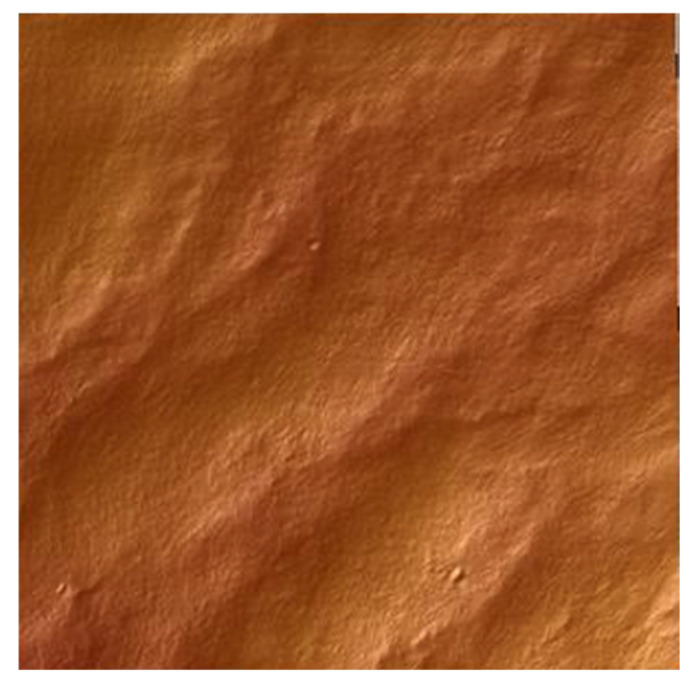
AFM visualization of collagen film.

**Figure 5:**
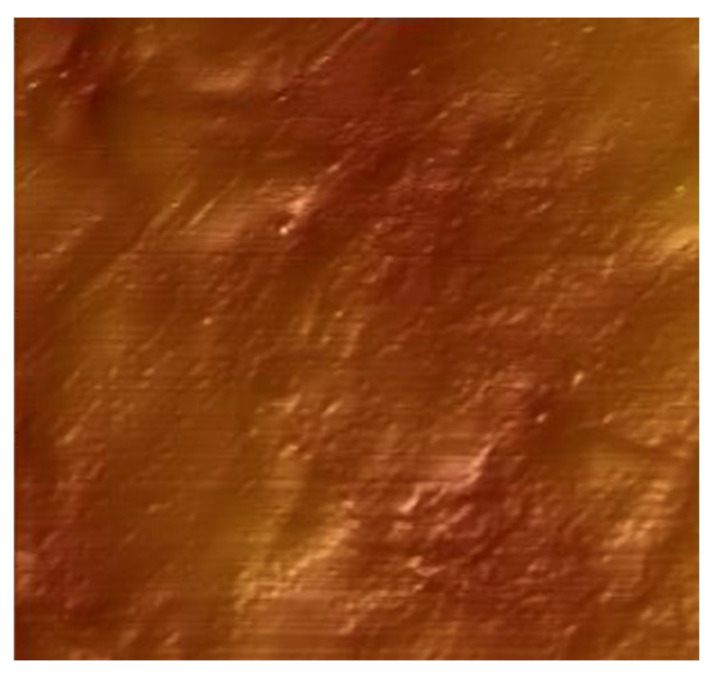
AFM visualization of salicin incorporated collagen film.

Atomic Force Microscopy (AFM) was carried out to evaluate the topography and surface structure of the material. AFM images have been shown in Figure 4 and Figure 5.

Root mean square average of height deviations (Rq) taken from the mean image data plane for each film was calculated by the pattern:

(1) $$\sqrt{\frac{\sum zi^{2}}{N}}$$

The value of *Rq* for collagen film was 172.05 nm, whereas for salicin-incorporated collagen film was 215.75 nm. The arithmetic average (*Ra*) of the absolute values of the surface height deviations measured from the mean plane for both films was also calculated:

(2) $$Ra=\frac{1}{N}\overset{N}{\underset{j=1}{\sum }}\left|Zj\right|$$

Values of *Ra* for collagen film and collagen film with salicin were 205.9 nm and 172.4 nm, respectively.

It was alleged that the addition of salicin modifies the superficial properties of collagen films. The roughness of collagen films changes significantly as a result of salicin addition.

### 3.4. Contact Angle Measurements

Contact angle measurements give information about hydrophobicity or hydrophilicity of the surface of materials. This property is very important when we consider the material application and decide about adhesion. Incorporating salicin into the collagen films has an impact on the contact angle. For collagen film, the contact angle for glycerin was 64.2° whereas for diiodomethane it was 47.4°. For collagen/salicin film the contact angle for glycerin was 69.3° whereas for diiodomethane 49.2°. The addition of salicin into collagen films influenced the increase in the contact angle. The calculated surface free energy (mJ/m^2^) was 38.5 for collagen films and 36.31 for collagen/salicin films. The polar component of surface free energy for collagen film was 10.07, whereas for collagen/salicin film it was 7.60. The addition of salicin to collagen leads to some decrease in hydrophilicity. The surface of the human skin in normal condition is generally hydrophobic because the outermost layer of skin, the stratum corneum, is composed of corneocytes and an intercellular lipid matrix. The decrease in hydrophilicity shows that for collagen/salicin film we can expect better adhesion to the human skin than for collagen films.

### 3.5. Mechanical Properties

Tensile strength of collagen and salicin-incorporated collagen films were tested (Table 2). The arithmetic mean from 7 samples was properly assessed for collagen film: 41.7 MPa and salicin-incorporated collagen film: 60.7 MPa, which indicates that the addition of salicin enhances the mechanical properties of collagen films. The above results may suggest the interactions between collagen and salicin. In fact, infrared spectroscopy showed the_shift of the amide A band in collagen after the addition of salicin (Table 1). The increase in mechanical properties is the second prove of interactions between collagen and salicin.

Young modulus was also examined, the results are shown in Table 2. The mean average for collagen film was 0.627 GPa, whereas for salicin-incorporated collagen film 1.42 GPa. This shows that tensile stiffness is greater in collagen film with the addition of salicin. The increase of mechanical properties of collagen films after salicin addition may suggest that the cross-linking process occurs.

### 3.6. Steady Shear Flow Properties

**Figure 6:**
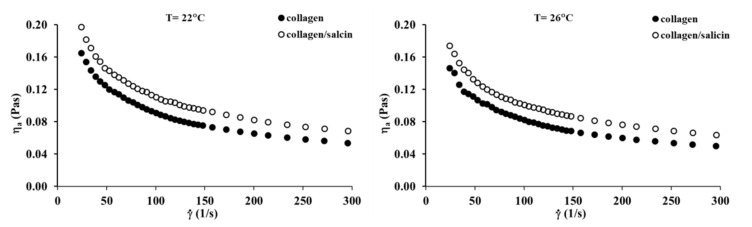
Viscosity curves of the collagen solutions at 22 °C and 26 °C.

The viscosity curves of the collagen and collagen/salicin solutions at 22 °C and 26 °C are presented in Figure 6. The apparent viscosity of collagen solutions decreases with increasing shear rate, in which typical shear-thinning behavior is observed. The addition of salicin to the collagen solution causes a marked increase in apparent viscosity. This can be attributed to interactions between the active groups of collagen and salicin. These results are consistent with infrared spectroscopy which indicates that the formation of new interactions between collagen molecules and salicin affected the apparent viscosity of the polymer solution.

**Figure 7:**
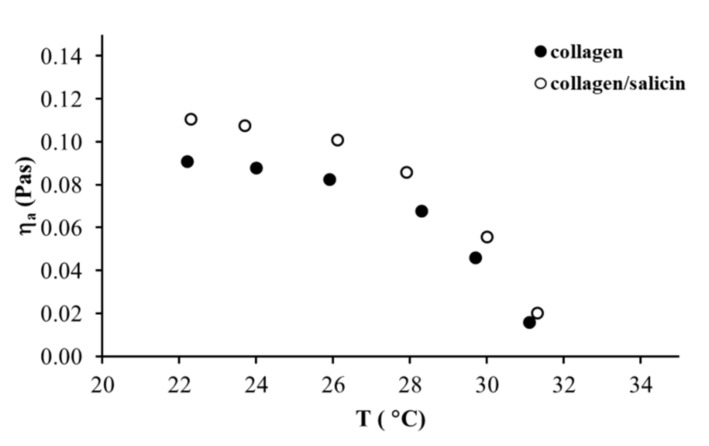
Apparent shear viscosity versus temperature for collagen solutions at $\dot{\gamma }$ = 100 s^−1^

The effect of temperatures on the apparent viscosity of the collagen and collagen/salicin solutions is shown in Figure 7. As can be observed, both collagen solutions exhibited a weak effect of temperature on the apparent viscosity (decreased slightly) in the temperature range between 22 °C and 28 °C. Further temperature increase from 29 °C to 31 °C induced a large reduction of apparent viscosity. These results indicated that collagen molecules were fully denatured above 31 °C. The addition of salicin to the collagen solution did not change the temperature of the denaturation of collagen molecules.

Steady shear flow properties of collagen solutions with the addition of salicin were well characterized by Ostwald de Waele model and Cross model. The rheological parameters calculated by these models are tabulated in Table 3. It can be noticed that the *n* parameter from the Ostwald de Waele model is below one, indicating shear-thinning behavior. Both *n* and *k* parameters are practically constant after the addition of salicin to the collagen solution and did not change with temperature increase. In the case of *η*_0_ and *η*∞ parameters from the Cross model, the parameter values increase after the addition of salicin. It is well known that the *η*_0_ parameter is directly associated with the number of interactions between the polymer macromolecules and other molecules in the solution [37,41,42]. Therefore, the increase in the *η*_0_ values after the addition of salicin confirms that the formation of new interactions between collagen molecules and salicin in the solution.

## 4. Discussion

**Figure 8:**
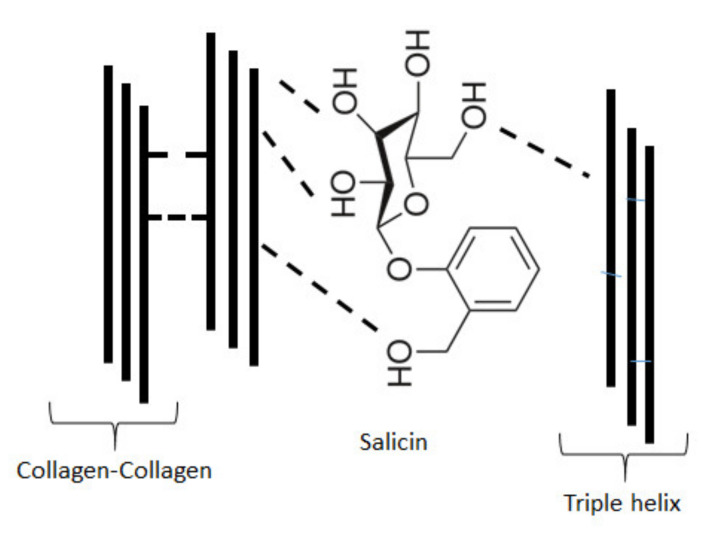
Examples of possible hydrogen bonds between collagen and salicin.

Collagen can be obtained from several animal tissues, however, the use of mammalian collagen had to be reduced because of the risk of bovine spongiform encephalopathy (BSE) and transmissible spongiform encephalopathy (TSE) development. Fish collagen is a safer alternative than collagen from mammalian tissues. Especially collagen extracted from marine sources such as fish skin, scales and bones, marine sponges, jellyfish umbrellas or starfish, is widely studied [20,21,36,42,43,44]. It has been shown that in cosmetic preparations, mammalian collagen can be successfully replaced with marine sponge collagen. The potential of marine collagen is rapidly growing. Fish collagen is commonly applied in food production and can replace mammalian collagen, but it has one disadvantage, low denaturation temperature. The low denaturation temperature of fish collagen is a result of the significantly lower content of hydroxyproline in the polypeptide chain of fish collagen than in the chain of mammalian collagen. Collagen extracted from silver carp skin has attracted much attention in recent years due to its relatively high denaturation temperature in comparison to collagen from other fish species [36]. Although there are some papers regarding the properties of collagen from silver carp, to our best knowledge, the influence of salicin on the rheological and film-forming properties of such kinds of collagen has not been studied yet. Collagen films were prearranged with Willow bark extract which contains 5% of salicin intending to ameliorate properties for cosmetic or biomedical applications. The results showed that the addition of salicin to the collagen solutions increases the apparent viscosity of a solution. It could be caused by interactions between the active groups of collagen and salicin. However, it should be emphasized that the rheological behavior of collagen solution depends also on concentration and temperature [43,44]. The results obtained by viscometry are coherent with infrared spectroscopy results which denote that new interactions between collagen molecules and salicin can modify the apparent viscosity of polymer solution. The incorporation of salicin into collagen film improved also the mechanical properties of the material. Tensile strength and Young Modulus for salicin-incorporated collagen films were significantly higher than those for collagen film. The possible interactions between collagen and salicin can be via hydrogen bonds. Salicin is an aryl β-d-glucoside and constitutes an aromatic primary alcohol-salicyl alcohol that has the phenolic hydrogen replaced by a β-d-glucosyl residue [40]. In salicin molecule, there are several OH groups from sugar ring which can form hydrogen bonds with several groups in collagen. Interactions between collagen and several polysaccharides via hydrogen bonds have been found previously [45,46]. In collagen molecule as well as in salicin molecule several chemical groups can form hydrogen bonds. The network of hydrogen bonds may increase both, rheological and mechanical properties of collagen. These properties may further improve the material adhesion to the skin. The examples of possible hydrogen bonds between collagen and salicin have been shown in Figure 8.

Collagen extracted from silver carp skin can be applied in many fields, such as food, pharmaceutical, cosmetic, biomedical materials, photographic gelatin, and leather industries [36,47]. Silver carp is one of the major species produced in Asia, especially in China, but nowadays it is also widely produced in many other countries. Fish skin is a byproduct of fishery processing and any ideas to improve or modify collagen properties extracted from such skin may lead to higher-value products from the processing of wastes. Combining properties of naturally occurring willow bark extract and collagen extracted from fish skin can allow for the production of new materials for contact with human skin. Contact angle measurements proved, that the adhesion of collagen films to human skin can be enhanced by the addition of salicin.

## 5. Conclusions

Willow bark extract containing salicin can be incorporated into collagen solutions and films. The addition of salicin to the collagen solutions increases its apparent viscosity. The results showed that the addition of salicin has also an enhancing influence on the film properties. Tensile strength of salicin-incorporated collagen films is significantly higher than in collagen film, likewise tensile stiffness. The addition of salicin modifies the superficial properties of collagen films. The surface roughness was bigger for collagen with salicin addition than in collagen films. The improvement of mechanical and surface properties may be due to hydrogen bonding between collagen and salicin molecules. The addition of salicin to collagen film may cause the improvement of the material adhesion to the skin, which can be a valuable feature in biomaterial application as well as anti-inflammatory properties of salicin. Further research is required to study biological activity and the cosmetic potential of salicin incorporated collagen films.

## Figures and Tables

**Table 1:** Wavenumbers for proper bonds in exact types of chemical compounds in collagen film.

IR Band	Stretching	Band Position for Collagen (cm^−1^)	Band Position for Collagen/Salicin (cm^−1^)
amide A	N-H, OH	3291	3297
amide I	C=O	1631	1631
amide II	N-H	1541	1540
amide III	C-N	1233	1234
glucoside	C-O-C	-	1027

**Table 2:** Mechanical properties of collagen and salicin-incorporated collagen film.

Material	F_max_ (MPa)	E_mod_ (GPa)
Collagen	41.7	0.627
Collagen/Salicin	60.7	1.42

**Table 3:** The Rheological Parameters from Ostwald de Waele Model and Cross Model for Collagen Solution and Collagen/Salicin Mixture.

T (°C)	Ostwald de Waele Model				Cross Model				
	*n*	*k* (Pas*^n^*)	*R* ^2^		*η*_0_ (Pas)	*η*∞ (Pas)	λ (s)	m	*R* ^2^
22	0.55	0.72	0.999	Collagen	0.369	0.00524	0.167	0.64	1.00
26	0.57	0.59	0.999		0.174	0.0321	0.00729	1.20	0.996
22	0.58	0.74	0.999	Coll/SL	0.613	0.00572	0.443	0.49	0.999
26	0.61	0.62	0.999		0.496	9.54 × 10^−4^	0.390	0.50	0.999

## Sample Availability

Samples of the compounds are not available from the authors.
